# Comparing Fidelity Outcomes of Paraprofessional and Professional Delivery of a Perinatal Depression Preventive Intervention

**DOI:** 10.1007/s10488-020-01022-5

**Published:** 2020-02-21

**Authors:** Alicia Diebold, Jody D. Ciolino, Jessica K. Johnson, Chen Yeh, Jackie K. Gollan, S. Darius Tandon

**Affiliations:** 1 Institute for Public Health and Medicine, Center for Community Health, Feinberg School of Medicine, Northwestern University, 750 N. Lake Shore Drive, Suite 643, Chicago, IL 60611 USA; 2 Division of Biostatistics, Department of Preventive Medicine, Feinberg School of Medicine, Northwestern University, Chicago, USA; 3 Department of Psychiatry and Behavioral Sciences, Feinberg School of Medicine, Northwestern University, Chicago, USA; 4 Department of Medical Social Sciences, Institute for Public Health and Medicine, Center for Community Health, Feinberg School of Medicine, Northwestern University, Chicago, USA

**Keywords:** Perinatal depression, Fidelity, Adherence, Competency

## Abstract

Mothers and Babies (MB), a perinatal depression preventive intervention, has proven effective in decreasing depressive symptoms and preventing onset of major depression. An ongoing cluster-randomized trial is comparing the effectiveness of the six-session MB group intervention led by paraprofessionals versus mental health professionals. Twenty percent of all audio-recorded intervention sessions were randomly selected for fidelity checks. Analyses assessed mean facilitator adherence and competency overall, by study arm, and by session; and, examined site, facilitator, and client-specific effects. There were no significant differences found between study arms. Findings show paraprofessionals can deliver MB with similar fidelity as mental health professionals.

**Electronic supplementary material:**

The online version of this article (10.1007/s10488-020-01022-5) contains supplementary material, which is available to authorized users.

## Background

In recent years, there has been a global push for using lay health workers to meet the growing health and mental health needs in underserved communities (Barnett et al. 2018). Lay health workers (referred to here as paraprofessionals) can provide services at a lower cost and assist in removing barriers to service delivery (Boer et al. 2005; Montgomery et al. 2010; Barnett et al. 2018). Additionally, paraprofessionals can aid in reducing the stigma of receiving mental health services by being able to offer culturally-appropriate services in agencies where people are already receiving other types of health and social services (Montgomery et al. 2010; Barnett et al. 2018).

One intervention that is examining paraprofessional delivery of mental health content is the Mothers and Babies (MB) Program. MB is a cognitive behavioral therapy (CBT)-based intervention that has been identified by the United States Preventive Services Task Force (2019) as an efficacious perinatal depression preventive intervention. Perinatal depression affects up to one in seven women in the United States during pregnancy and the first year after birth (Gavin et al. 2005) and can have negative impacts on both mother and child (O’Hara and McCabe 2013). The group modality of MB has proven effective in reducing depressive symptoms and preventing the onset of new cases of perinatal depression when delivered by a mental health professional (MHP) (McFarlane et al. 2017; Tandon et al. 2013; Le et al. 2011; Muñoz et al. 2007). A current study is building on previous MB research by determining if paraprofessionals can be effective in preventing the onset and worsening of depressive symptoms when delivering MB.

There have been several reviews examining studies that have compared professional and paraprofessional delivery of interventions to treat depression and anxiety. A review conducted by Boer et al. (2005) focused solely on randomized controlled trials (RCT) comparing the effectiveness of interventions led by paraprofessionals and professionals to treat anxiety and depression in adults. Montgomery et al. (2010) focused specifically on CBT interventions delivered by paraprofessionals and professionals to treat anxiety and depression. Studies identified in both reviews were a mixture of either group or individual delivery. These reviews found little difference between patient outcomes regardless of who led the intervention. However, very few of the studies included in these reviews reported on implementation outcomes, such as fidelity of intervention delivery. Fidelity measures the extent that the intervention was delivered as intended (adherence) and how skillfully it was delivered (competence) (Waltz et al. 1993). Assessing fidelity is imperative in understanding intervention outcomes (Weck et al. 2011; Waltz et al. 1993), as both adherence and competence can provide useful information to help guide conclusions about intervention effectiveness, or lack thereof. Beyond assessment of fidelity, going a step further to identify variables that can influence fidelity can be of benefit to researchers and practitioners (Boswell et al. 2013).

Data reported here come from a cluster-RCT studying the comparative effectiveness of MHPs and paraprofessionals in delivering a group version of MB (Jensen et al. 2018). Primary results for this study on preventing the onset of major depression and worsening of depressive symptoms are forthcoming. Since the previous studies of the MB group modality centered on the use of MHPs, further research is needed to assess how well paraprofessionals can deliver the intervention. Therefore, an additional aim of the trial assessed the fidelity of group facilitators delivering MB. Johnson et al. (2019) details how fidelity was measured [through the modification of the Revised Cognitive Therapy Rating Scale (Blackburn et al. 2001) resulting in the MB-CTS], and how inter-rater reliability was established and maintained among coders prior to assessment of facilitator fidelity. The current manuscript builds on this previous work and has two objectives: (1) To determine whether adherence and competency outcomes differ by study arm (paraprofessional vs. MHP) and/or intervention session; and, (2) To evaluate the influence of site, facilitator, and client characteristics on adherence and competency.

## Methods

### Mothers and Babies Intervention

Mothers and Babies (MB) is a perinatal depression preventive intervention that can be delivered with a woman 1-on-1 or in a group setting. The group modality is delivered over the course of six weeks in sessions that last between 60–120 min. The content of MB is guided by CBT principles of monitoring and scheduling pleasant activities (Sessions #1 & #2), evaluating the validity of thoughts and developing strategies for reframing harmful thought patterns (Sessions #3 & #4), and developing strong support systems (Sessions #5 & #6). Each session consists of several topics, with an instructor manual that provides a script and key points to aid in the delivery of the intervention (Segovia et al., n.d). There is also a participant manual with activities for participants to complete during, and after, sessions (Degillio et al., n.d).

### Study Participants

A total of 37 home visiting (HV) programs participated in the research study, located in one of seven states: Illinois, Ohio, Iowa, Michigan, Minnesota, Missouri, and West Virginia. Criteria for program selection included the ability to recruit a large number of pregnant clients in a 16–18 month period. Programs were randomized into one of three study arms: (1) MB group intervention led by paraprofessional home visitors; (2) MB group intervention led by MHPs; or, (3) control (see Jensen et al. 2018, for more information on the randomization process). Clients were recruited through HV programs and the surrounding communities to participate in the research study. Enrollment criteria for clients included: being ≥ 16 years old; being ≤ 33 weeks gestation at time of referral; and speaking either English or Spanish. In total, 874 pregnant women were recruited, with 715 women enrolled in one of the intervention arms, and 538 receiving at least one MB session. The racial/ethnic make-up of participants was: 44.8% Black/African American, 30.1% white, 19.5% Hispanic/Latina, 3.9% bi-racial, 1.0% Asian, and 0.7% Native American.

Paraprofessional home visitors were recruited through HV programs randomized to the study arm assigned to deliver MB groups using paraprofessionals. These paraprofessionals were employed as staff at the HV program and were either self-selected or selected by a supervisor. MHPs were recruited either through the HV programs (if an existing staff member met eligibility criteria) or through professional associations (e.g., a state infant mental health association). The research team assisted in identifying a MHP to facilitate groups if a program had difficulty in finding someone who met the qualifications. In total, 53 facilitators were recruited, trained, and delivered the intervention (32 paraprofessional home visitors and 21 MHPs). Criteria for paraprofessional facilitators included not having advanced degrees beyond a bachelor’s degree in mental health or a related field; there was no minimum degree requirement. Criteria for MHP facilitators included holding an advanced degree (master’s or beyond) in a related field and five years’ experience with children and/or families. Two exceptions were made for MHP facilitators who did not have a master’s degree but had over 10 years’ experience and some master’s coursework completed. All facilitators across study arms were female and the racial/ethnic breakdown of the facilitators was: 53.2% white, 23.4% Black/African American, and 23.4% Hispanic/Latina. Ten facilitators (four MHPs and six paraprofessional home visitors) delivered groups in Spanish. The rest of the groups were delivered in English.

Prior to implementing the MB group intervention, facilitators received 8–12 h of training from the study Principal Investigator (PI). The same training content was provided to facilitators regardless of study arm. Training was conducted via one of the following modalities:

1. In person
2. Live webinar
3. Previous training on the 1-on-1 version of MB followed by a call with the PI to discuss differences with the group model
4. Recording of a live training followed by a call with the PI to review and answer any questions

The PI provided supervision for facilitators, over the phone in a group setting, during a facilitator’s implementation of her first cohort (six group sessions). Fidelity checks reflect sessions facilitated by 48 of the 53 facilitators. Table 1 shows characteristics of these 48 facilitators.

**Table 1:** Facilitator characteristics

	Paraprofessional-led		Mental health professional-led		All	
Characteristic	n	%	n	%	N	%
	29	60.42	19	39.58	48	100.00
Education level						
Less than master's degree	24	82.76	2	10.53	26	54.17
Master's degree or Above	5	17.24	17	89.47	22	45.83
Experience working/practicing in the field of early childhood						
1–2 years	11	37.93	2	10.53	13	27.08
3–5 years	8	27.59	3	15.79	11	22.92
6–10 years	4	13.79	5	26.32	9	18.75
11–15 years	4	13.79	3	15.79	7	14.58
> 15 years	2	6.90	6	31.58	8	16.67
Knowledge of cognitive behavioral theory and/or attachment theory						
None	4	13.79	–	–	4	8.33
Some	13	44.83	2	10.53	15	31.25
Moderate	12	41.38	13	68.42	25	52.08
Expert	–	–	4	21.05	4	8.33
Previous experience leading groups						
None	3	10.34	–	–	3	6.25
Some	18	62.07	9	47.37	27	56.25
A lot	8	27.59	10	52.63	18	37.50
Training modality						
In person	26	89.66	8	42.11	34	70.83
Live webinar	–	–	6	31.58	6	12.50
1-on-1 MB training + call	2	6.90	2	10.53	4	8.33
Recording of in-person training + call	1	3.45	3	15.79	4	8.33

*N* = 48. Facilitator characteristics are represented for 48 individual facilitators captured in the 160 sessions included in the fidelity checks

### Data Collection

The research study and all materials were approved through the Northwestern University Institutional Review Board (STU00203761). All facilitators consented to participate in the research study. After enrollment, facilitators were asked to complete a brief survey that included questions regarding demographic information (race/ethnicity, educational level achieved), previous work experience (number of years in the field), previous experience with research (none, some, moderate, or expert), groups (level of experience leading), and/or MB (read/heard about it, attended a training, and/or used the curriculum), and extent of previous knowledge of CBT and/or attachment theory (none, some, moderate, or expert). All data was collected and stored in a secure web application, Research Electronic Data Capture (REDCap) (Harris et al. 2009).

Group sessions took place from January 2017 to October 2018. Each session was audio-recorded and uploaded to a secure server. Twenty percent of all completed sessions were randomly selected for fidelity coding. Randomization involved generating a random number for each possible recorded session at each site, sorting the observations in ascending order by that random number, and preserving the lowest 20% as those to be selected for coding. Two coders from outside the research team (both with advanced training in psychology) were trained on the MB group intervention and the fidelity process. The coders established sufficient inter-rater reliability (IRR) on six unique study tapes prior to individual coding. Sufficient IRR was defined as above 90% adequate agreement (within one point) on the competency items. Adequate agreement was achieved on 96% of the items (95% confidence interval [CI]: 88%, 99%) in these six sessions (Johnson et al. 2019). Twelve additional sessions were coded by both coders throughout the coding process to ensure IRR was maintained (for more information on the training, coding, and IRR process, see Johnson et al. 2019).

The coders used a modified version of the Cognitive Therapy Scale-Revised (CTS-R, Blackburn et al. 2001) for assessing adherence and competency. The CTS-R is based on the original Cognitive Therapy Rating Scale (Young and Beck 1980), and contains 12-items used to measure a therapist’s competence in delivering cognitive therapy. The modified CTS-R maintained most of the general competency items, adapted the more technical competency items for use with the MB group intervention, and added an “Engagement” item for measuring the facilitator’s ability to engage all of the women in the group. More specific detail around adherence to the intervention was included in the beginning of the rating scale in the form of a checklist for completion of topics within the MB curriculum (see Johnson et al. 2019, for more information on the development of this tool). Facilitators were rated on a three-point scale (“Not covered at all,” “Partially covered,” “Completely covered”) for adherence of each topic within a session. Competency was assessed for each session in 12 areas and rated on a six-point scale ranging from “Poor” to “Excellent” (see Johnson et al. 2019 for an example). Competency scores of three or greater indicated satisfactory competency (Johnson et al. 2019, Young and Beck 1980). Ratings were entered and stored in REDCap (Harris et al. 2009).

## Analysis

Primary outcomes for analyses included a mean percent adherence score (the average adherence divided by the total possible score) and a mean competency rating (across all 12 items) for each session. Both were treated as continuous measures for analyses. We further explored individual competency item scores. To accomplish primary objectives, we examined a number of potential predictors/covariates. They included: randomization arm (MHP vs. paraprofessional), site, facilitator identifier, session number, population density of the site, percent of participants identifying as a racial/ethnic minority at that session, average baseline depressive symptom score for participants within the session, participant count in a given session, facilitator demographic variables (education level, years working in the field, previous knowledge of CBT, experience leading groups), race concordance (if the facilitator’s race matched the majority race of clients) within a group for a given session, and facilitator training modality (see Online Appendix A for the full Statistical Analysis Plan).

We calculated descriptive statistics for all variables of interest. Categorical variables were summarized with frequencies and percentages, and continuous variables were summarized with means and standard deviations or medians and interquartile ranges, as appropriate. Boxplots and histograms, as appropriate, were used to illustrate outcome distributions across arms, sessions, education level, etc.

Primary analyses utilized separate linear mixed models (LMMs) to examine average competency score and average adherence by study arm and session number. Specifically, models for each outcome included a fixed effect for either arm or session (depending upon hypothesis of interest) and a random facilitator effect to account for the same facilitators within arm and sessions. We also considered inclusion of a random site effect to account for differences between sites. However, including a random site effect and a random facilitator effect resulted in an overly saturated model in which both random effects were not estimable. Thus, for analyses in general, we deemed it sufficient to include a single random facilitator effect.

Secondary analysis further used separate LMMs to evaluate influence of site, participant, and facilitator characteristics on outcomes of average competency score and average adherence. As above, the models contain a fixed effect for site, participant or facilitator characteristics (one-at-a-time in individual models) and a random facilitator effect. Exploratory analysis used the same analytic strategy to evaluate influence of exploratory variables such as the size of group, and facilitator demographics and experience on these outcomes. Exploratory analyses also examined individual competency score items (dichotomized into an indicator variable based on a score of at least ‘3′, which reflected “satisfactory” competency) via a series of generalized LMMs with logit link and binomial distributional assumption. We evaluated predictive ability of study arm, session, and race concordance in these models. The models had a fixed effect on the exploratory variable and a random effect on either facilitator or site, as appropriate. Since the individual items are scored on a Likert scale, often with “ceiling” or “flooring” effects, we a priori chose to dichotomize the individual item scores for analyses. This allowed us to explore behavior of individual items in relation to these potential predictors rather than overall score.

As the fidelity analyses are ancillary to a larger cluster-randomized trial with a pre-specified analysis plan, they are more exploratory in nature and, thus, there were no formal power calculations in evaluating this aim. All analyses assumed a two-sided 5% level of significance. If any individual, multi-level variable was significant at the 5% level, we used a Tukey adjustment for pairwise comparisons to further explore any additional contrasts. All analyses were conducted in SAS version 9.4 (The SAS Institute; Cary, NC).

## Results

**Fig. 1:**
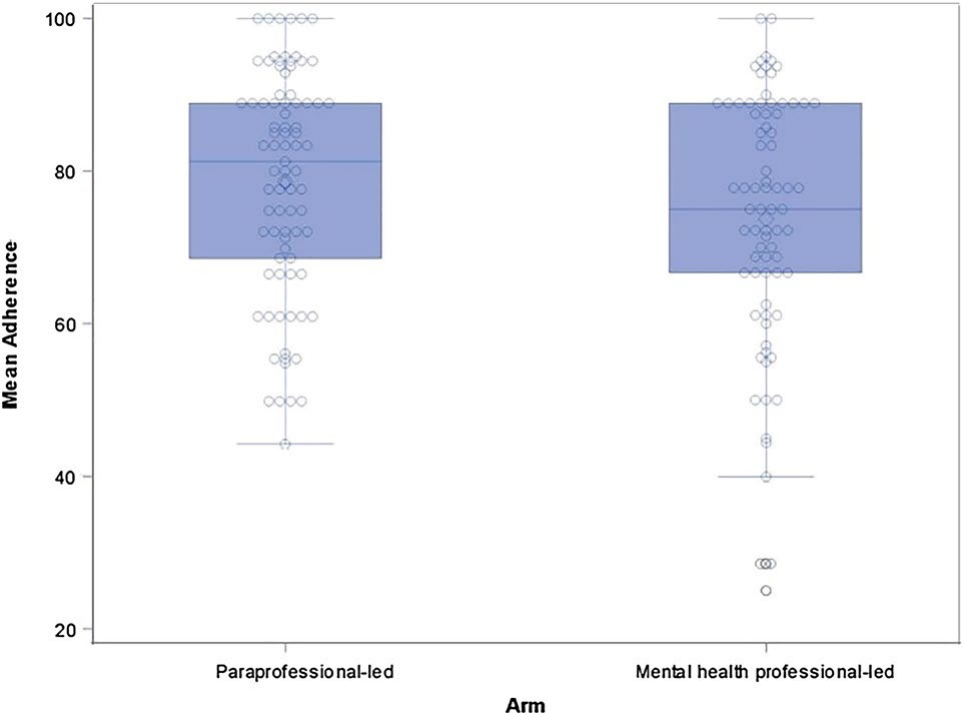
Mean adherence by arm. This is a boxplot representing the mean adherence scores for the two study arms, paraprofessional-led and MHP-led. Analysis did not identify a significant difference between arms

**Fig. 2:**
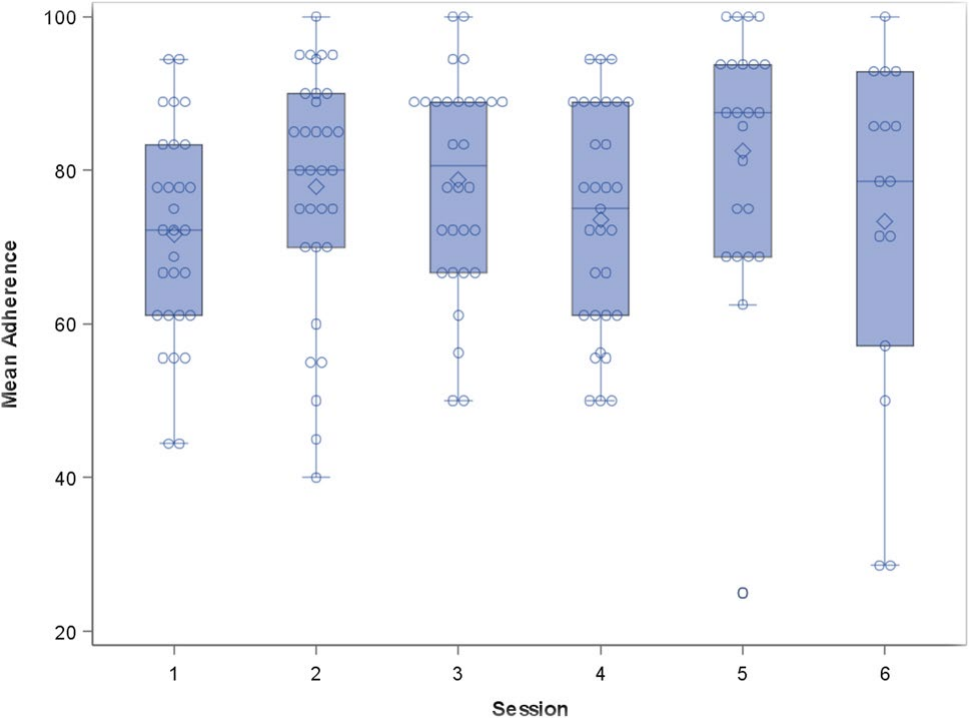
Mean adherence by session. This is a boxplot representing the mean adherence scores for each session (1–6). There was a significant session effect found in the pairwise comparison for Session #3 vs Session #4

Analyses included a total of 160 rated sessions. Table 2 shows mean outcome scores overall for average adherence and competency, and by individual competency item. The mean adherence score was 76.32% and overall mean competency score was 3.76 (slightly above “satisfactory”). Neither arm nor session were significantly associated with mean competency (p = 0.512 and p = 0.533, respectively). Although arm was not significantly associated with adherence score (p = 0.090, see Fig. 1), there was a significant session effect on adherence (p = 0.009, see Fig. 2). After adjusting for multiple hypothesis tests, just one of the pairwise comparisons remained significant: Session #3 [model-estimated mean ± standard error (SE) = 81.2 ± 2.79] vs. Session #4 [71.2 ± 2.79; Tukey-adjusted mean difference = 10.0 with 95% confidence intervals (CI) 0.3–19.7; adjusted p = 0.040], meaning there was greater adherence of Session #3 compared to Session #4.

**Table 2:** Descriptive statistics for overall mean adherence scores, competency scores, and individual competency items

	Mean	SD	Median	Q1	Q3
Average adherence score	76.32	16.00	77.78	66.67	88.89
Mean competency score	3.76	0.64	3.83	3.25	4.25
Agenda setting and adherence	2.78	1.33	3.00	2.00	4.00
Feedback	3.39	0.87	3.00	3.00	4.00
Understanding	3.94	0.72	4.00	4.00	4.00
Interpersonal effectiveness	4.34	0.72	4.00	4.00	5.00
Collaboration	3.73	0.86	4.00	3.00	4.00
Pacing and efficient use of time	3.38	1.12	4.00	2.00	4.00
Emotional expression	4.01	0.82	4.00	4.00	4.00
Guided discovery	3.43	0.94	3.00	3.00	4.00
Focusing on key thoughts or behaviors	3.75	0.89	4.00	3.00	4.00
Application of Mothers and Babies techniques	3.66	1.08	4.00	3.00	5.00
Personal projects	3.38	1.06	3.00	3.00	4.00
Engagement	5.29	0.91	6.00	5.00	6.00

*SD* standard deviation, *Q1 *first quartile, *Q3 *third quartile

**Fig. 3:**
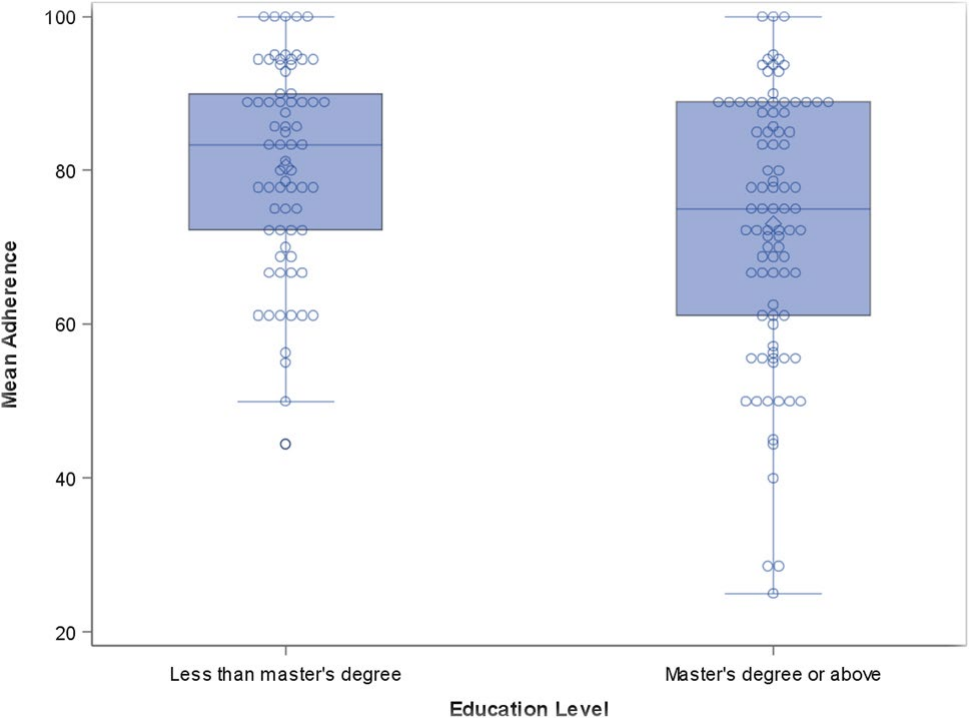
Mean adherence by facilitator education level. This is a boxplot representing the mean adherence scores by facilitators’ education level. Mean adherence for facilitators with less than a master’s degree was significantly higher than those with a master’s degree or above

**Fig. 4:**
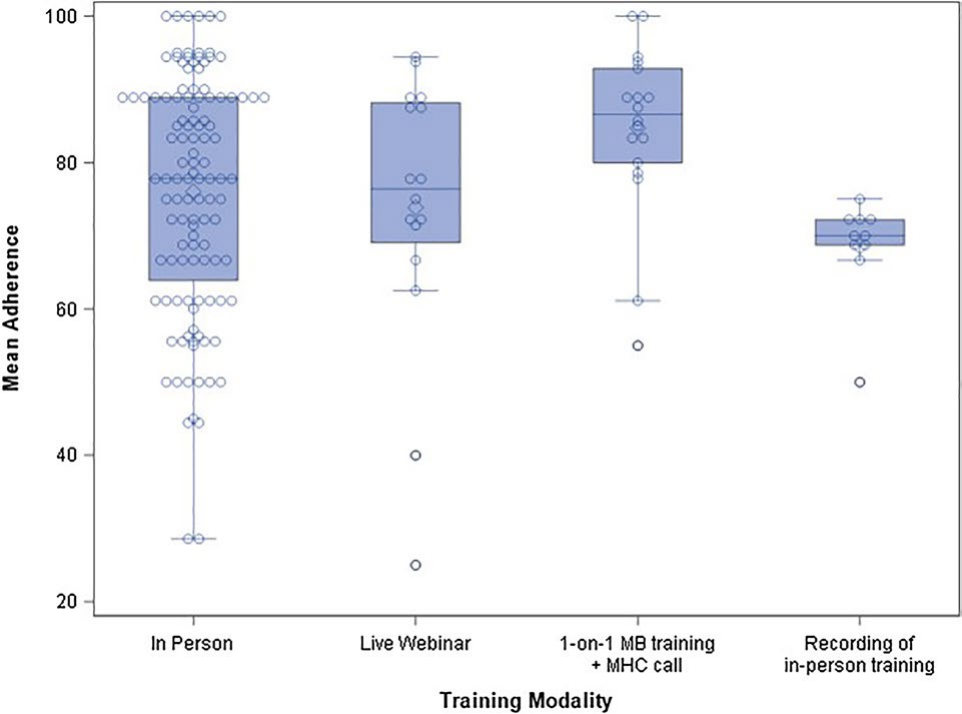
Mean adherence by training modality. This is a boxplot representing the mean adherence scores by facilitator training modality. Mean adherence for facilitators trained previously on MB 1-on-1 was significantly higher than those trained via a recording of a live training

Upon examination of site, facilitator, and client-specific effects, results were largely non-significant with a few exceptions. Education level (two-level factor) was significantly associated with adherence (p = 0.038, see Fig. 3). Those with less than a master’s degree tended to show better adherence than those with a master’s degree or higher (model-estimated difference = 7.5 with 95% CI 0.5–14.5; p = 0.038). Training modality was also significantly associated with adherence (overall p = 0.047, see Fig. 4). After adjustment for multiple comparisons, the pairwise comparison that remained significant was that between 1-on-1 MB training followed by a call on the group model vs. recording of in-person training (model-estimated difference = 18.7 with 95% CI 0.2–37.2; p = 0.046). Facilitators who were trained on the 1-on-1 modality of MB followed by a call had significantly greater adherence than facilitators who were trained by listening to a recording of an in-person training.

Race concordance between facilitator and the group was not significantly associated with the outcomes in this dataset. Exploratory analyses examining arm and session on individual competency items were non-significant except for one item. Competency Item #1: Agenda Setting and Adherence was significant (p = 0.033) between Session #1 and Session #2, with facilitators in Session #1 having significantly greater competency on Item #1 than Session #2.

## Discussion

The aim of this manuscript was to assess fidelity of the MB group modality via adherence and competency, comparing differences by study arm and session number, and exploring potential facilitator, client, and site-level variables that may influence these outcomes. Overall, analyses suggest no significant differences between paraprofessionals and MHPs for either their overall adherence to delivering the MB intervention or their competency in delivering the intervention. Moreover, we do not have evidence of significant differences between paraprofessionals and MHPs on any individual competency item.

Facilitators with a master’s degree or above had overall lower average adherence than those with less than a master’s degree. Facilitators with higher education, particularly related to mental health, may have had more confidence delivering the curriculum’s CBT-based content giving them increased comfort in going off script more often than facilitators who may have less familiarity with the CBT concepts. Aarons et al.’s (2011) Exploration, Preparation, Implementation, and Sustainment (EPIS) conceptual framework for examining intervention implementation suggests that varying clinician attitudes toward evidence-based interventions may be another possible explanation for our finding that MHPs had lower adherence when delivering MB. For example, some studies have found that MHPs may find evidence-based interventions too restrictive in meeting clients’ varied needs irrespective of how long the MHP had worked in the field as a clinician (Brookman-Fazee et al. 2016; Burgess et al. 2016). While most previous research has examined attitudes of MHPs toward evidence-based interventions, Jensen-Doss et al. (2009) examined attitudes of both MHPs and providers with less formal education. Their study found that MHPs with more mental health training had less positive attitudes toward evidence-based treatments.

Similarly, lower average adherence was found for facilitators who were trained via listening to a recording of a live training than facilitators who were trained previously on the 1-on-1 modality of MB plus a supplemental call with the PI on the differences of the group modality. Bryan et al. (2009) note that one key adult learning principle that may affect success in training public health practitioners is that learners are actively involved in the learning process. Facilitators who listened to the recording were given an opportunity to ask questions and review the material with the PI; however, this did not provide the same degree of interactions with the PI and peers as the in-person trainings, which may have impacted the learning process. This finding highlights an important consideration for future MB intervention trials as well as other intervention research when considering ways in which facilitator training is conducted.

Across all competency items, “Engagement” had the highest mean score among both types of facilitators. This exemplifies facilitators’ abilities to get clients involved in the discussion and participate in activities. This high rating could be a reflection of the previous experience facilitators had leading groups, as only 6% had no prior experience leading any type of group. Many paraprofessionals employed by health and social service agencies who may potentially serve as intervention facilitators have existing relationships with clients at their agencies. These existing relationships could explain the high “Engagement” scores found in this study and suggest that other studies using paraprofessionals may similarly find strong engagement between facilitators and intervention participants. The competency item “Agenda Setting and Adherence” had the lowest mean score. We believe it is likely that facilitators prioritized agenda setting—which occurred at the beginning of each session—less than other things that also needed to be accomplished at the start of each group, including checking in with group members about their psychological status. Facilitators were encouraged during their training to be responsive to personal (e.g., psychological, financial) issues that clients may be dealing with during group sessions, which may have led to frequent deviations from the way a session agenda was presented in the instructor manuals. This focus on attending to clients’ personal issues is a hallmark of community health workers, particularly paraprofessional home visitors (Korfmacher 2016), who are encouraged to be client-centered in their approach.

### Strengths and Limitations

Our rigorous study design for assessing fidelity, including audio-recording each session, utilizing multiple coders, and establishing IRR, was a major strength of this work. In addition to initially establishing IRR, our ongoing assessment of IRR and re-training, as needed, was also a strength (Johnson et al. 2019). One limitation for this study included not capturing unique sessions. Some facilitators had to combine sessions (most commonly Sessions #5 & #6) due to agency and client needs. Since this would have created complications for the coders, these sessions were eliminated from being scored. In the future, it may be helpful to score these sessions to see if there is any variation in fidelity when sessions are combined. A second limitation was the number of statistical hypothesis tests. While we used Tukey’s adjustment for multiple pairwise post hoc comparisons for potential predictors with more than two categories, we evaluated 11 different potential predictors for each outcome and the number of tests performed nonetheless increased the risk of making a type I error. Foreseeing this issue, we developed an analysis plan (see Online Appendix A) a priori to document and outline the statistical hypothesis tests and to prevent too many ad hoc analyses. Lastly, the small sample size, especially in training modality subgroups, presents another limitation for examining subgroup differences in study outcomes. Note that although statistically significant differences in adherence scores between some subgroups were observed, we caution the reader that these results cannot be deemed confirmatory as these mean estimates may be unstable. We further caution the reader that while there were largely insignificant findings in comparing paraprofessional home visitors to MHPs, we cannot claim that the two are equivalent as this was not designed as a formal equivalence study. The failure to reject our null hypothesis simply means that in this dataset we do not have evidence against the claim that the outcome scores, on average, are equal across the two groups.

## Conclusions

Findings of this study suggest that paraprofessionals can deliver a CBT intervention with a comparable level of fidelity as MHPs, adding to the literature that they may be a feasible and cost-effective alternative for delivering CBT in underserved communities. Our findings have significant implications for the adoption and sustainment of evidence-based interventions in the context of HV programs. Although HV models that use professionals (e.g., social workers, nurses) exist, most HV models employ paraprofessionals. Our findings suggest that HV programs should feel comfortable in having paraprofessional home visitors—trained on MB—deliver the intervention to clients. The variables that appear preliminarily associated with adherence and competency will also guide the researchers in improving language and structure of the MB instructor manual, and future trainings and supervision of facilitators.

## Electronic supplementary material

Below is the link to the electronic supplementary material.

Electronic supplementary material 1 (DOCX 28 kb)
