# Supplementary material for: Comparing Fidelity Outcomes of Paraprofessional and Professional Delivery of a Perinatal Depression Preventive Intervention
Source: Adm Policy Ment Health. 2020 Feb 21;47(4):597–605. doi: 10.1007/s10488-020-01022-5 (PMC7253394; doi:10.1007/s10488-020-01022-5)
Supplement: Supplementary file 1 — Electronic supplementary material 1 (DOCX 28 kb) [file 10488_2020_1022_MOESM1_ESM.docx]

**STATISTICAL ANALYSIS OUTLINE – Fidelity Outcomes for a Group-Based Intervention Led by Paraprofessionals and Professionals to Prevent Postpartum Depression**

1. **INTRODUCTION**

This document will outline the statistical analysis plan (SAP) for a secondary analysis of the study: Comparing the Effectiveness of Clinicians and Paraprofessionals to Reduce Disparities in Perinatal Depression. The parent study is a Patient-Centered Outcomes Research Institute (PCORI) – funded, three-arm, cluster-randomized study evaluating efficacy of the Mothers and Babies (MB) course when delivered by paraprofessional home visitors (HV) in comparison to mental health (MH) professionals. The details of the primary analyses for this study are specified elsewhere. The purposes of the analyses specified in this document, we will only focus on the two active intervention arms (HV and MH).

1. **ANALYSIS OBJECTIVES**
   1. Assess overall fidelity via competence and adherence scores.
   2. Determine whether fidelity outcomes differ by:
      1. Arm (facilitator type: MH / HV).
      2. Session number (#1, #2, …, #6). There are a total of six sessions involved in the MB Course.
   3. Evaluate the influence of agency / participant / facilitator characteristics on competency and adherence, including:
      1. Population density at the site. This information may be obtained from the randomization table data as this variable was included in the randomization algorithm.
      2. Race (% minority for the session). This information will come from the participant (main) database.
      3. Participants’ mental health at baseline. This information will also come from the main database. We will measure mental health via the Quick Inventory of Depressive Symptoms (16-item self-report survey; QIDS).
   4. Exploratory objectives include evaluating the effect of the following variables on adherence and competence.
      1. Size of group during a given session.
      2. Facilitator demographics / level of experience:
         1. Education level.
         2. Years in field.
         3. Previous knowledge on cognitive behavioral therapy (CBT) / attachment therapy.
         4. Experience leading groups.
         5. Race concordance with group.
         6. Training modality: in-person, webinar, one-on-one training and MHC call, recording of in-person training.
2. **STUDY DESIGN/SAMPLE**

**2.1. Study Type –** This is a secondary analyses from the MB cluster-randomized study, evaluating fidelity at the session level through a random sample of recorded and scored MB sessions.

**2.2. Sample Size –** N=160 recorded sessions (random sample of sessions) among the two intervention arms only (MH and HV).

1. **OUTCOMES**
   1. Mean competency score – This will be treated as continuous measure as it is the mean value of 12 competency items (each scored 0-6).
   2. Individual competency item score – Each of the individual items will be analyzed / summarized separately as either categorical / ordinal variables as the competency is assessed on a 0 to 6 ordinal scale.
   3. Average adherence score – We plan to treat this outcome as continuous; however, we will examine distributional characteristics prior to analyses to determine the appropriate methods for analyses. Adherence items are given a score of 10 (completely covered), 5 (partially covered), 0 (not covered at all). The average adherence score is calculated as a mean of 10 topics (items), multiplied by 100.
2. **COVARIATES/CONFOUNDERS**
   - Arm (MH vs. HV) will be treated as a fixed effect.
   - Site will be considered a potential random effect.
   - Facilitator ID may be added as a random effect.
   - Session number will be treated as a fixed effect.
   - Population density of site (from randomization) will be treated as a fixed effect.
   - Percent minority at session will be treated as a fixed effect. We will calculate this value via mapping the race for participants in session from the main database to the session data.
   - Mental health will be treated as a fixed effect and measured via the QIDS. We will again calculate this value via mapping the relevant fields from the main database to the fidelity outcomes database.
   - Participant count in a given session. This variable comes from the fidelity dataset (the sign-in form) and it was added post hoc.
   - Facilitator demographic variables (these come from main database in Arm #5): education level, years in field, previous knowledge of CBT / attachment therapy, experience leading groups.
   - Race concordance with group for a given session: if > 50% of group members’ races = that of the facilitators (minority vs. non-minority). We will also use 2x2 table(s) to explore the concordance / discordance in general.
   - Training modality: in-person, webinar, one-on-one training and MHC call, recording of in-person training. These data come from a separate database that will be merged with the analytic data file.
3. **STATISTICAL ANALYSIS PLAN**
   1. *GENERAL CONSIDERATIONS*

General analytic strategy: generalized linear (mixed) model(s) (GLMMs) for each outcome vs. primary predictor(s) of interest. Initially, we plan to use normal theory methods, and in the case of violations of assumptions, we may consider nonparametric methods or transformation of variables. Generalized linear models will involve either identity link with normal distributional assumptions (continuous outcome measures) or logit link with binomial distribution assumptions (binary outcome). If outcomes must be considered ordinal, we will explore either cumulative logistic regression or multinomial logistic regression (generalize logit link function). Since the aims of these analyses are not meant to develop a predictive model for outcomes, but to examine the association between a series of predictors and outcomes, we will explore each potential predictor’s or covariate’s association with each outcome one-at-a-time via inclusion of a fixed / random effect as indicated above. The exception may be the inclusion of random effects to account for repeated measures within facilitator / site; in these cases, if these effects are significant, we will include these random effects in each GLMM. As these analyses are purely exploratory in nature, we do not plan to account for multiple hypothesis tests. We also anticipate the analyses changing as we explore distributional and statistical assumptions.

- 1. *DATA ANOMALIES*

There are multiple sessions that were assessed by more than one rater. We will randomly sample one of those sessions and use that session for analyses. We are confident in doing so because we were able to establish sufficient interrater agreement (Jensen et al. 2019; under review).

In addition, we will exclude ID 132 (Tape ID 1038109435) and ID 79 (Tape ID 101142425) from analysis: ID 132 had two sessions combined and an incomplete recording, and ID 79 was incomplete recording.

- 1. *PLANNED ANALYSES*
     1. **Aim: Assess overall fidelity via competence and adherence**

Descriptive statistics will summarize the overall competence and adherence outcomes. Categorical variables will be summarized with use of counts and percentages, and continuous variables with means and standard deviations or median and interquartile range as appropriate.

- - 1. For all additional analyses, refer to the table below and the general analytic strategy outlined above for the planned modeling techniques.

| Predictor | Variable Type | Outcome | GLMM Type | Fixed Effect | Random Effect |
| --- | --- | --- | --- | --- | --- |
| **2.2 Arm/ Session** | | | | | |
| Arm | Category | Mean competency score | Generalized linear models with identity link | Arm | Facilitator |
|  |  | Individual item score | Generalized linear models with logit link  Cumulative logistic regression | Arm | Facilitator |
|  |  | Average adherence score | Generalized linear models with identity link | Arm | Facilitator |
| Session | Category | Mean competency score | Generalized linear models with identity link | Session | Facilitator |
|  |  | Individual item score | Generalized linear models with logit link  Cumulative logistic regression | Session | Facilitator |
|  |  | Average adherence score | Generalized linear models with identity link | Session | Facilitator |
| **2.3 agency / participant / facilitator characteristics** | | | | | |
| Population density at the site | Continuous | Mean competency score | Generalized linear models with identity link | Population density | Facilitator |
|  |  |  |  |  |  |
|  |  | Average adherence score | Generalized linear models with identity link | Population density | Facilitator |
| Race (% minority for the session) | Ordinal / Continuous | Mean competency score | Generalized linear models with identity link | Percent Minority | Facilitator |
|  |  |  |  |  |  |
|  |  | Average adherence score | Generalized linear models with identity link | Percent Minority | Facilitator |
| Participants’ mental health | | | | | |
| Some variation of QIDS score (mean or median from the group score at baseline) | Continuous | Mean competency score | Generalized linear models with identity link | Mean or median QIDS | Facilitator |
|  |  |  |  |  |  |
|  |  | Average adherence score | Generalized linear models with identity link | Mean or median QIDS | Facilitator |
| **2.4 Exploratory** | | | | | |
| Participant count | Continuous | Mean competency score | Generalized linear models with identity link | Count | Facilitator |
|  |  |  |  |  |  |
|  |  | Average adherence score | Generalized linear models with identity link | Count | Facilitator |
| Facilitator demographics / level of experience | | | | | |
| Education level | Category | Mean competency score | Generalized linear models with identity link | Education level | Site |
|  |  |  |  |  |  |
|  |  | Average adherence score | Generalized linear models with identity link | Education level | Site |
| Years in field | Continuous | Mean competency score | Generalized linear models with identity link | Years in field | Site |
|  |  |  |  |  |  |
|  |  | Average adherence score | Generalized linear models with identity link | Years in field | Site |
| Previous knowledge on cognitive behavioral therapy (CBT) / attachment therapy | Category | Mean competency score | Generalized linear models with identity link | Previous knowledge | Site |
|  |  |  |  |  |  |
|  |  | Average adherence score | Generalized linear models with identity link | Previous knowledge | Site |
| Experience leading groups | Category | Mean competency score | Generalized linear models with identity link | Experience | Site |
|  |  |  |  |  |  |
|  |  | Average adherence score | Generalized linear models with identity link | Experience | Site |
| Race concordance with group | Category | Mean competency score | Generalized linear models with identity link | Concordance | Site |
|  |  | Individual item score | Generalized linear models with logit link  Cumulative logistic regression | Concordance | Site |
|  |  | Average adherence score | Generalized linear models with identity link | Concordance | Site |
| Training modality | Category | Mean competency score | Generalized linear models with identity link | Modality | Site |
|  |  |  |  |  |  |
|  |  | Average adherence score | Generalized linear models with identity link | Modality | Site |

- - 1. **Exploratory Analyses**

We will explore the relationship between predictors via a series of bivariate analyses (chi-squared, scatter plots, etc.). The details of these will be outline after the initial analyses are completed above.

- 1. *TECHNICAL*
     1. All analyses will be conducted using SAS version 9.4 (Cary, NC).
